# Astroglial reactivity is a key modulator of Alzheimer’s disease pathological progression

**DOI:** 10.1093/brain/awae354

**Published:** 2024-11-08

**Authors:** Wiesje Pelkmans, Juan Domingo Gispert

**Affiliations:** Barcelonaβeta Brain Research Center (BBRC), Pasqual Maragall Foundation, 08005 Barcelona, Spain; Hospital del Mar Medical Research Institute (IMIM), 08003 Barcelona, Spain; Barcelonaβeta Brain Research Center (BBRC), Pasqual Maragall Foundation, 08005 Barcelona, Spain; Hospital del Mar Medical Research Institute (IMIM), 08003 Barcelona, Spain; Universitat Pompeu Fabra, 08002 Barcelona, Spain; Centro de Investigación Biomédica en Red de Bioingeniería, Biomateriales y Nanomedicina (CIBER-BBN), 28029 Madrid, Spain; Centro Nacional de Investigaciones Cardiovasculares (CNIC), 28029 Madrid, Spain

## Abstract

This scientific commentary refers to ‘Association of glial fibrillary acid protein, Alzheimer's disease pathology and cognitive decline’ by Peretti *et al*. (https://doi.org/10.1093/brain/awae211).

**This scientific commentary refers to ‘Association of glial fibrillary acid protein, Alzheimer's disease pathology and cognitive decline’ by Peretti *et al*. (https://doi.org/10.1093/brain/awae211).**

Neuroinflammation plays an increasingly recognized role in the pathophysiology of Alzheimer's disease. Astrocytes and microglia in the brain become activated in response to amyloid-β (Aβ) plaques and tau tangles, and this activation is now understood to drive pathological progression in Alzheimer’s disease, rather than being merely a bystander effect.^1^ In light of this, inflammatory markers, albeit not specific to Alzheimer’s disease, have been included in the revised National Institute on Aging and Alzheimer’s Association (NIA-AA) criteria for Alzheimer's disease diagnosis and staging, alongside amyloid, tau, and neurodegeneration.

Among these neuroinflammatory markers, glial fibrillary acidic protein (GFAP) has emerged as a sensitive and robust biomarker for astrocyte reactivity.^2^ It can be measured in both CSF and serum, although its levels in these fluids may differ markedly, for reasons that remain unclear.^3^ Notably, plasma GFAP levels are significantly elevated in association with early amyloid pathology.^4^ Even in cognitively unimpaired individuals, plasma GFAP may impact the aggregation of fibrillar Aβ^5^ and the phosphorylation of tau.^6^ These findings suggest a key role for astrogliosis in very early Alzheimer's disease pathogenesis, with elevated plasma GFAP detectable decades before the onset of symptoms. GFAP levels continue to rise across the Alzheimer's disease continuum, providing robust prognostic information on the risk of progression to dementia.^2^ Plasma GFAP is also being used as a pharmacodynamic marker in clinical trials of anti-Aβ immunotherapies, where its levels drop with treatment-driven reductions in amyloid plaques and clinical benefit.

However, important questions remain about how GFAP interacts with Alzheimer's disease pathology in more advanced disease stages. Conflicting evidence exists: while some studies show stronger associations between plasma GFAP and amyloid, others link plasma GFAP more closely with tau pathology, or suggest that GFAP is independently linked to both amyloid and tau. A ‘two-wave’ model of astrocyte reactivity has been proposed to reconcile these apparent discrepancies. According to this model, a first wave of astrocyte reactivity occurs during presymptomatic or prodromal stages, and a second wave occurs at more advanced stages of Alzheimer's disease dementia.^7^

In this issue of *Brain*, Peretti and co-workers^8^ investigate the relationship between plasma GFAP levels, Aβ and tau pathology, and cognitive decline in a memory clinic cohort. The study included 122 participants across the Alzheimer's disease clinical spectrum [cognitively unimpaired, mild cognitive impairment (MCI), dementia], classified according to their amyloid-tau (AT) profiles. Plasma GFAP levels were found to correlate with several key Alzheimer's disease markers: higher amyloid PET Centiloid levels, higher global and regional Braak (I–V) tau standardized uptake value ratios (SUVRs), lower cortical thickness in signature areas, and worse Mini-Mental State Examination (MMSE) scores at baseline and over time. Even after adjusting for age, sex, education, and APOE ε4 carriership, higher plasma GFAP levels remained significantly associated with cognitive decline and elevated global tau SUVRs. Notably, these associations were independent of amyloid load. Plasma GFAP was also found to partially mediate the relationship between global Aβ PET and global tau PET uptake, as well as between tau PET and MMSE decline. However, no significant associations were found between global Aβ PET Centiloid and GFAP when correcting for tau load and demographic factors. Furthermore, plasma GFAP did not mediate the relationship between Aβ PET and the MMSE annual rate of change.

Peretti and co-workers’ study^8^ confirms that astrocyte reactivity, as indicated by GFAP levels, significantly contributes to amyloid-induced tau pathology. These findings align with recent research suggesting that serum GFAP is more strongly associated with tau tangles than amyloid plaques in individuals with advanced Alzheimer's disease pathology,^9^ indicating that astrocytes may become primarily reactive to tau pathology in later disease stages.

On the other hand, prior research has consistently demonstrated a strong link between plasma GFAP and amyloid levels. In the current study, the fact that this association did not survive after correcting for tau suggests that the study may have predominantly captured the ‘second wave’ of astrocyte activation, when amyloid biomarkers approach a plateau (Fig. 1). In this phase, astrocyte reactivity may play a key role in determining whether individuals with amyloid deposits progress to downstream tau pathology, neurodegeneration, and cognitive decline.

**Figure 1:**
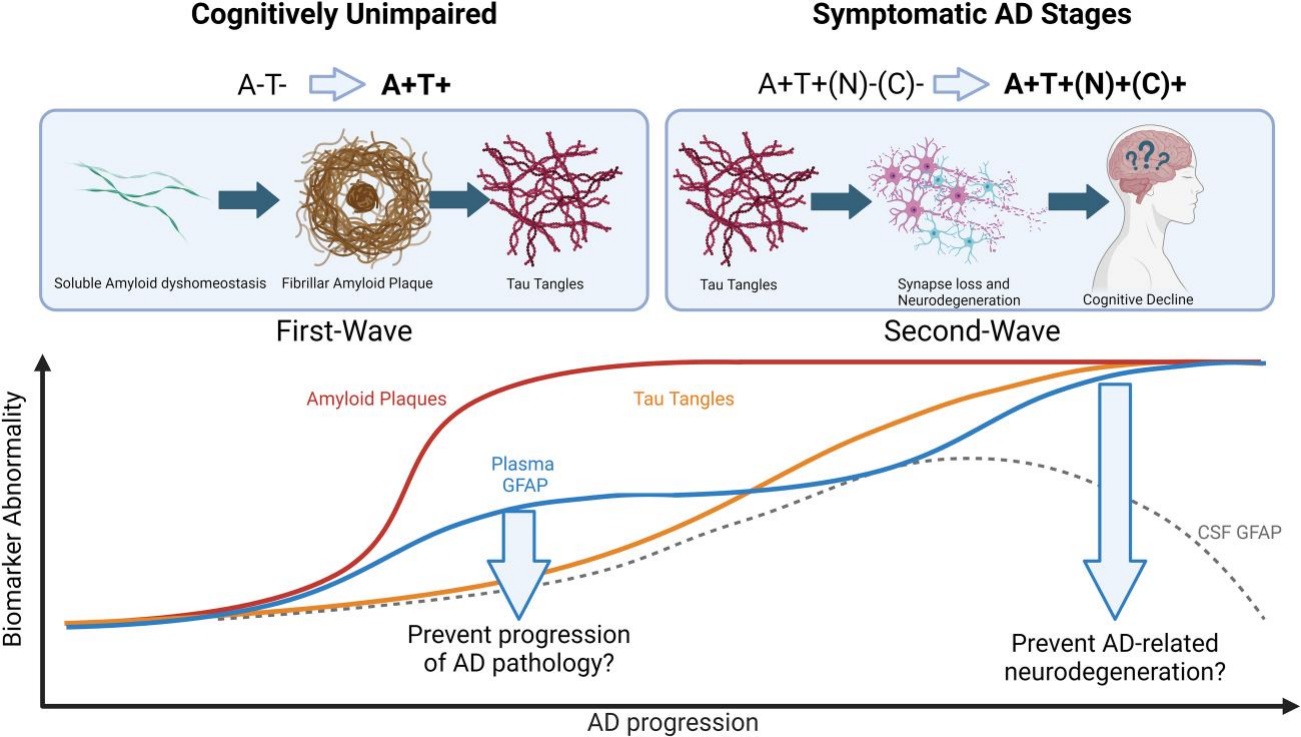
**Hypothetical course of plasma GFAP concentrations over the course of Alzheimer’s disease progression according to the ‘two-wave’ model of astrocyte reactivity**. In the first wave, plasma GFAP rises early in the Alzheimer’s disease (AD) continuum, in association with soluble amyloid-β (Aβ) dyshomeostasis, and may play a key role in fibrillary Aβ aggregation and tau phosphorylation. At later stages of the Alzheimer’s disease pathophysiological cascade, plasma GFAP may influence Aβ-driven aggregation of tau in neurofibrillary tangles. Created in BioRender.com.

Longitudinal studies are now required to better characterize the dynamic relationship between GFAP and Alzheimer's disease pathology. Such research could help resolve the conflicting findings on the role of GFAP throughout the disease continuum. In addition, there is a need to better understand the pathological processes driving elevated plasma GFAP concentrations and whether these levels accurately reflect GFAP expression globally across the brain or only within certain subpopulations of astrocytes.^9^ PET ligands, such as ^18^F-SMBT-1 and ^18^F-DED, can provide valuable insights into regional astroglial reactions to Alzheimer's disease pathology. Of particular interest would be to assess astrogliosis in relation to varying regional patterns of tau tangle spread, which may offer a more nuanced understanding of downstream neurodegenerative and cognitive consequences.

Associations between GFAP and cerebral glucose consumption are also intriguing. Plasma GFAP has been found to be positively associated with increased ^18^F-FDG PET uptake in the brains of cognitively unimpaired individuals with reduced (i.e. more pathological) CSF Aβ_42/40_ levels. On the other hand, in amyloid and CSF p-tau_181_ positive individuals, CSF GFAP is negatively associated with ^18^F-FDG PET uptake in brain regions that typically exhibit glucose hypometabolism associated with neurodegeneration in Alzheimer’s disease.^10^ Future research should also explore other astroglial biomarkers, such as YKL-40 and S100B, as well as microglial markers like sTREM2 in conjunction with GFAP. The distinct dynamics displayed by these markers across Alzheimer’s disease stages may provide insights into different phenotypes of glial reactivity and, therefore, help to capture different aspects of neuroinflammation involved in Alzheimer’s disease pathological progression.

Ultimately, interventional studies will be needed to investigate whether targeting astroglial pathways can modify the progression of Alzheimer’s disease pathology. To this end, convenient, sensitive, and robust biomarkers will be required to identify the right individuals at the right stage for intervention, as well as to demonstrate target engagement.^1^ At present, plasma GFAP appears to be a leading candidate biomarker for these purposes.

The study by Peretti *et al.*^8^ underscores the evolving understanding that neuroinflammation is not merely a bystander, but a driving force of Alzheimer's disease progression. By identifying plasma GFAP as a key modulator of this process, the study adds to the groundwork required to develop novel therapeutic approaches for Alzheimer’s disease that extend beyond the traditional focus on amyloid and tau. As we advance towards targeted stage-specific therapies, the potential for personalized treatments becomes more tangible. While the road to understanding and combating Alzheimer’s disease remains long, each discovery brings us closer to a future where we can slow, halt, or even prevent this devastating disease, offering hope to millions of patients and their families.
